# The burden of influenza A and B in Mexico from the year 2010 to 2013: An observational, retrospective, database study, on records from the Directorate General of Epidemiology database

**DOI:** 10.1080/21645515.2018.1456281

**Published:** 2018-05-10

**Authors:** Ricardo Cortes-Alcala, Gaël Dos Santos, Rodrigo DeAntonio, Raghavendra Devadiga, Cuitlahuac Ruiz-Matus, Maria E. Jimenez-Corona, Jose A. Diaz-Quinonez, Luis Romano-Mazzotti, Maria Yolanda Cervantes-Apolinar, Pablo Kuri-Morales

**Affiliations:** a GSK, Calz México-Xochimilco 4900, Col. San Lorenzo Huipulco, Mexico City, Mexico; b GSK, Av. Fleming 20, 1300 Wavre, Belgium; c GSK, Urbanización Industrial Juan Díaz Entre Calles A y B, Apartado Postal 6-1697, Panama City, Panama; d GSK, 5, Embassy Links, SRT Road, Opp to Accenture, Cunningham Road, Vasanth Nagar, Bengaluru, Karnataka, India; e Director General of Epidemiology, Ministry of Health, Francisco de P. Miranda 177 Lomas de Plateros, Ciudad de México, México; f Deputy Director General of Epidemiology, Ministry of Health, Francisco de P. Miranda 177 Lomas de Plateros, Ciudad de México, México; g Deputy Director General of the Institute for Epidemic Diagnose and Reference, Ministry of Health, Francisco de P. Miranda 177 Lomas de Plateros, Ciudad de México, México; h Faculty of Medicine, National Autonomous University of Mexico, Division of Graduate Studies, Avenida Universidad 3000, Copilco El Bajo, Coyoacan, CDMX, Ciudad de México, México; i 5, Crescent Dr, Philadelphia, Philadelphia, PA, USA; j GSK, Calz México-Xochimilco 4900, Col. San Lorenzo Huipulco, Ciudad de México, México; k Assistant Secretary for Health Promotion and Disease Prevention, Lieja No. 7, Col. Juarez, Ciudad de México, México

**Keywords:** Epidemiology, Influenza A, Influenza B, Influenza vaccine, Mexico, Surveillance

## Abstract

Despite vaccination programs, influenza still represents a significant disease burden in Mexico. We conducted an observational, retrospective analysis to better understand the epidemiological situation of the influenza virus in Mexico. Analysis of the seasonal patterns of influenza A and B were based on the Directorate General of Epidemiology dataset of influenza-like illness(ILI), and severe acute respiratory infection(SARI) that were recorded between January 2010 and December 2013. Our objectives were 1) to describe influenza A and B activity, by age group, and subtype and, 2) to analyze the number of laboratory-confirmed cases presenting with ILI by influenza type, the regional distribution of influenza, and its clinical features. Three periods of influenza activity were captured: August 2010–January 2011, December 2011–March 2012, and October 2012–March 2013. Cases were reported throughout Mexico, with 50.3% (n = 10,320) of cases found in 18–49 year olds. Over the entire capture period, a total of 76,085 ILI/SARI episodes had swab samples analyzed for influenza, 27% were positive. During the same period, influenza A cases were higher in the 18–49 years old, and influenza B cases in both 5–17 and 18–49 age groups. Peak activity occurred in January 2012 (n = 4,159) and December 2012 (n = 348) for influenza A and B respectively. This analysis confirms that influenza is an important respiratory pathogen for children and adults in Mexico despite vaccination recommendations. School-age children and adolescents were more prone to influenza B infection; while younger adults were susceptible to both influenza A and B viruses. Over the seasons, influenza A and B co-circulated.

## Introduction

In 2009, Mexico experienced a severe influenza A (H1N1) outbreak, which was the premise for the first Public Health Emergency of International Concern (PHEIC) and according to the international health regulations the outbreak could be confirmed as a pandemic. This pandemic was characterized by high morbidity and mortality rates especially during its first outbreak, and there was an increase in high transmission rates between younger and relatively healthier individuals than previously observed.^1-4^

Vaccination against influenza began during the latter part of 2004 in children of 6 to 24 months of age, and adults 65 years and older. By winter 2006–2007 the vaccination age for children was extended to 35 months. This meant an increase from 89.8% to 91.7% of the target programmed by the Health Sector and Ministry of Health. The target of 100% was attained for people aged 60 and over.^5^

Prior to the 2009 influenza pandemic, the vaccination recommendation in Mexico was that the vaccine should contain two subtypes of influenza A virus and one subtype of influenza B virus and administered as an annual dose a few months before the start of winter. The recommendation was also that children should receive the fractionated vaccine. In addition, people 60 years and older and those at high risk should receive first preference for vaccination. High-risk patients include those with chronic diseases of the renal, pulmonary or cardiovascular system and metabolic diseases including anaemia, diabetes and immunosuppression.^6^

Important studies have been published on the specific characteristics and outcomes of this influenza pandemic and the subsequent seasonal outbreaks of influenza in Mexico.^1-3^^,^^7-11^ However, the patterns of influenza occurrence during the years following the pandemic infection have been poorly documented.

In 2006, the national surveillance program database at the Directorate General of Epidemiology (DGE), a sentinel platform to conduct influenza surveillance, was implemented in Mexico. Since then, all cases of influenza like-illness (ILI) and severe acute respiratory infection (SARI), have been registered via this platform in the DGE national surveillance system.^12^

This observational retrospective analysis was conducted to assess the burden of influenza in Mexico, the analysis involved describing the seasonal activity patterns of influenza A and B viruses, using the DGE dataset of ILI and SARI records collected between January 2010 and December 2013.

## Results

### Participants

**Figure 1.**
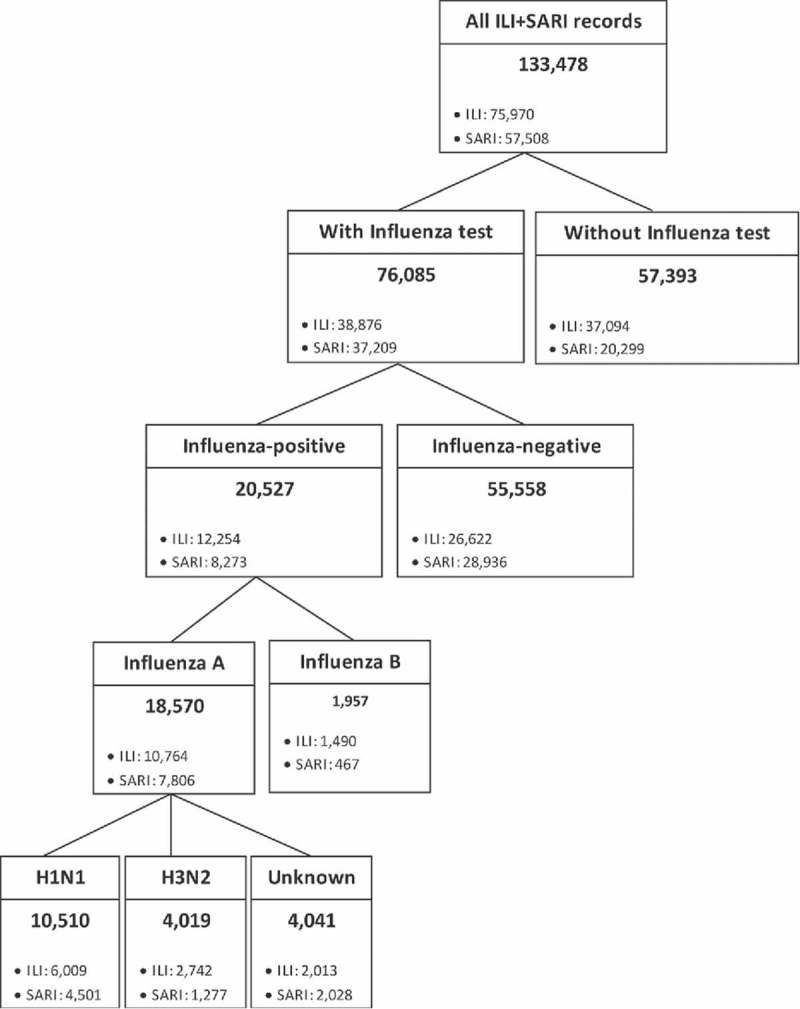
Classification of the population by influenza diagnosis and by ILI and SARI episodes, 2010–2013. Acronyms: ILI, influenza-like illness; SARI, severe acute respiratory infection.

A total of 133,478 episodes of ILI and SARI were reported between January 2010 and December 2013. Of these, 57% (n = 76,085) had samples tested for influenza, and among them 73% (n = 55,558) were negative (Figure 1). The classification of these episodes by influenza diagnosis and by ILI or SARI subgroup is illustrated in Figure 1.

Viewing data from laboratory tests, the percentage of influenza positivity ranged from 9.5% in 2011 to 34.4% in 2012 based on data by year and amongst the ILI and SARI patients (see Supplementary Table 1).

### Descriptive data

Half of the influenza-positive cases were in the age group 18–49 years (Table 1). One third of all influenza-positive cases were admitted to hospital as a consequence of their illness (Table 1).

**Table 1.** Summary of demographic characteristics by influenza status.

	Influenza-positive	Influenza-negative
	N = 20,527	N = 55,558
Sex, male, n (%)	9,578 (46.7)	26,995 (48.6)
Age, years^*^		
Median (range)	29.0 (0–111)	26.0 (0–109)
Age groups, n (%)		
0–4 years	2,618 (12.8)	16,990 (30.7)
5–17 years	3,728 (18.2)	5,779 (10.5)
18–49 years	10,320 (50.4)	19,076 (34.5)
50–64 years	2,540 (12.4)	6,005 (10.9)
≥65 years	1,256 (6.1)	7,442 (13.5)
missing	65 (0.3)	266 (0.5)
Region, n (%)		
Center	6,377 (31.1)	16,931 (30.5)
Center-West	4,442 (21.6)	12,913 (23.2)
North	4,963 (24.2)	10,110 (18.2)
South-Southeast	4,745 (23.1)	15,604 (28.1)
Patient type		
Ambulatory, n (%)	12,965 (63.2)	21,084 (37.9)
Hospitalized, n (%)	7,562 (36.8)	34,474 (62.1)

* age was available for 20,462 influenza-positive and 55,292 influenza-negative records.

N: Number of ILI+SARI records with samples tested for influenza.

n: number of records within given category.

Acronyms: ILI, influenza-like illness; SARI, severe acute respiratory infection.

Table 1 displays the main demographic characteristics of the population with influenza diagnosis (positive or negative). Table 2 displays the main demographic characteristics of analysis by diagnosis, influenza type and season stratified by season.

**Table 2.** Summary of outcome by diagnosis, influenza type and season.

		ILI				SARI				Total			
		Flu_A		Flu_B		Flu_A		Flu_B		Flu_A		Flu_B	
		N = 10764		N = 1490		N = 7806		N = 467		N = 7806		N = 467	
Season	Outcome	n	%	n	%	n	%	n	%	n	%	n	%
2010	Death	60	1.7	0	0.0	170	5.8	0	0.0	230	3.6	0	0.0
	Discharged	204	5.8	5	2.0	555	19.0	2	5.7	759	11.8	7	2.4
	Follow-up	773	22.1	43	16.9	42	1.4	5	14.3	815	12.7	48	16.6
	In Treatment	1595	45.5	156	61.4	1740	59.6	10	28.6	335	51.9	166	57.4
	Non severe cases	622	17.7	45	17.7	226	7.7	7	20.0	848	13.2	52	18.0
	Severe cases	249	7.1	4	1.6	177	6.1	11	31.4	426	6.6	15	5.2
	Transferred	2	0.1	1	0.4	8	0.3	0	0.0	10	0.2	1	0.3
	Missing	120	—	7	—	2	—	3	—	122	—	10	—
2011	Death	10	2.4	0	0.0	47	14.6	1	3.1	57	7.8	1	0.5
	Discharged	37	9.0	4	2.2	59	18.3	4	12.5	96	13.1	8	3.7
	Follow-up	79	19.3	49	26.6	2	0.6	2	6.3	81	11.1	51	23.6
	In Treatment	158	38.6	97	52.7	141	43.8	7	21.9	299	40.9	104	48.1
	Non severe cases	90	22.0	20	10.9	35	10.9	8	25.0	125	17.1	28	13.0
	Severe cases	35	8.6	14	7.6	34	10.6	10	31.3	69	9.4	24	11.0
	Transferred	0	0.0	0	0.0	4	1.2	0	0.0.	4	0.5	0	0.0
	Missing	7	—	8	—	0	—	0	—	7	—	8	—
2012	Death	59	1.4	1	0.1	256	9.4	6	2.7	315	4.5	7	0.8
	Discharged	262	6.0	20	2.9	749	27.4	47	21.0	1011	14.3	67	7.3
	Follow-up	1445	33.2	138	20.1	42	1.5	2	0.9	1487	21.0	140	15.4
	In Treatment	1926	44.3	436	63.4	1093	40.1	86	38.4	3019	42.7	522	57.2
	Non severe cases	490	11.3	76	11.0	333	12.2	52	23.2	823	11.6	128	14.0
	Severe cases	155	3.6	17	2.5	239	8.8	31	13.8	394	5.6	48	5.3
	Transferred	10	0.2	0	0.0	17	0.6	0	0.0	27	0.4	0	0.0
	Missing	206	—	3	—	7	—	0	—	213	—	3	—
2013	Death	79	3.6	4	1.2	245	13.4	6	3.5	324	8.1	10	1.9
	Discharged	249	11.5	33	9.5	519	28.4	40	23.1	768	19.2	73	14.1
	Follow-up	498	22.9	96	27.7	20	1.1	2	1.2	518	13.0	98	18.9
	In Treatment	1000	46.1	173	50.0	636	34.8	75	43.4	1636	40.9	248	47.8
	Non severe cases	238	11.0	25	7.2	279	15.3	31	17.9	517	12.9	56	10.8
	Severe cases	104	4.8	14	4.0	117	6.4	19	11.0	221	5.5	33	6.4
	Transferred	2	1	1	0.3	12	0.7	0	0.0	14	0.4	1	0.2

ILI = Episodes with influenza like illness.

SARI = Episodes with severe acute respiratory infection.

Flu_A = Episodes positive for Influenza A.

Flu_B = Episodes positive for Influenza B.

N = number of episodes.

n = number of episodes in a given category.

% = n / Number of episodes with available results × 100.

The monthly activity during 2010–2013 ranged between 2 to 4,159 cases (0.3–53.3% of tested samples), and from 0 to 348 cases (0%–21.8% of tested samples) for influenza A and B, respectively. The highest number of cases was recorded in January 2012 for influenza A, and in December 2012 for influenza B.

**Figure 2.**
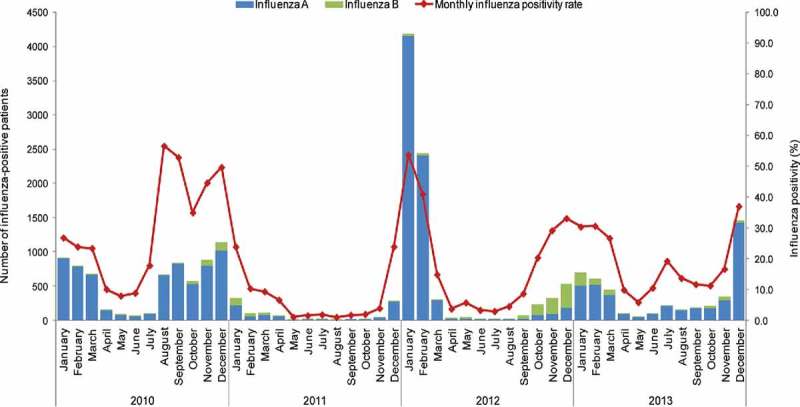
Seasonal pattern of laboratory-confirmed influenza A or B activity, 2010–2013.

In the 2010–2011 influenza season, the highest peak of influenza-positive patients was recorded in December 2010: 44.3% (n = 1,142) of the respective ILI+SARI patients based on available influenza laboratory test results. This influenza season lasted for six months, from August 2010 to January 2011 (Figure 2).

**Figure 3.**
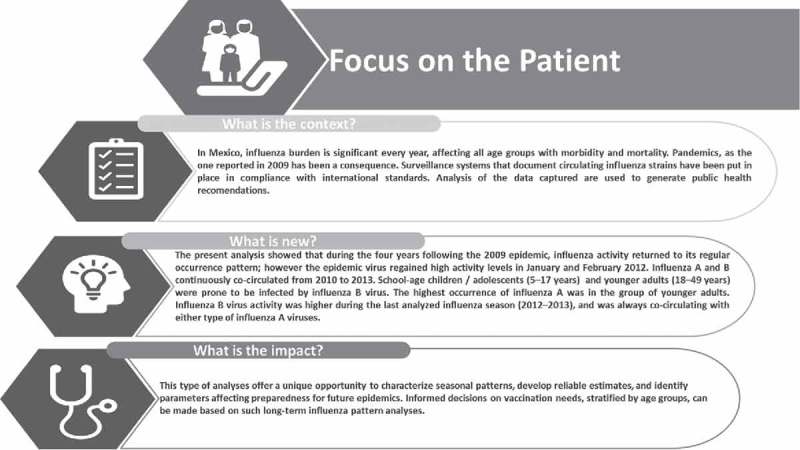
Summary of context, outcomes and impact for healthcare providers.

In the 2011–2012 influenza season, the highest peak of influenza-positive patients was recorded in January 2012: 53.7% (n = 4,189), of the respective ILI+SARI patients (based on available influenza laboratory test results). This influenza season lasted for four months, from December 2011 to March 2012 (Figure 2).

In the 2012–2013 influenza season, the highest peak of influenza-positive patients was recorded in January 2013: 30.3% (n = 695), of the respective ILI+SARI patients with available influenza laboratory test results. This influenza season lasted for six months, from October 2012 to March 2013 (Figure 2).

The highest activity for influenza B was reported during 2012–2013. Global Influenza Surveillance and Response System (GISRS) data show that the influenza B virus co-circulated with the A H3N2 virus until the 12th week of 2013.^13^ In the 13th week, influenza B co-circulated with the pandemic A (H1N1)pdm09 virus.^13^ Our dataset ended in December 2013, but the GISRS 2013–2017 data for Mexico showed that influenza B virus continued to co-circulate with either A subtype (A H3N2 or A (H1N1)pdm09).^13^

### Influenza cases characterisation

The age groups with the highest influenza occurrence were 18–49 years for all influenza A subtypes; and 5–17, and 18–49 years old for influenza B (Table 3). On average, 22% (n = 4,041) of all 2010–2013 influenza A cases were not subtyped. Lineage characterisation was not reported for the influenza B viruses.

**Table 3.** Occurrence of influenza A and B by age group, within all ILI+SARI records, 2010–2013.

		Influenza A							
	ILI+SARI records	All subtypes^*^		subtype H1N1		subtype H3N2		Influenza B	
	N	n	% (95%CI)	n	% (95%CI)	n	% (95%CI)	n	% (95%CI)
Overall	76,085	18,570	24.4 (24.1–24.7)	10,510	13.8 (13.6–14.1)	4,019	5.3 (5.1–5.4)	1,957	2.6 (2.5–2.7)
Age groups									
0–4 years	19,608	2,296	11.7 (11.3–12.2)	1,021	5.2 (4.9–5.5)	581	3.0 (2.7–3.2)	322	1.6 (1.5–1.8)
5–17 years	9,507	3,030	31.9 (30.9–32.8)	1,758	18.5 (17.7–19.3)	710	7.5 (6.9–8.0)	698	7.3 (6.8–7.9)
18–49 years	29,396	9,624	32.7 (32.2–33.3)	5,824	19.8 (19.4–20.3)	1,856	6.3 (6.0–6.6)	696	2.4 (2.2–2.5)
50–64 years	8,545	2,393	28.0 (27.1–29.0)	1,467	17.2 (16.4–18.0)	440	5.1 (4.7–5.6)	147	1.7 (1.5–2.0)
≥65 years	8,698	1,168	13.4 (12.7–14.2)	409	4.7 (4.3–5.2)	417	4.8 (4.4–5.3)	88	1.0 (0.8–1.2)
Years									
2010	21,911	6,545	29.9 (29.3–30.5)	2,202	10.0 (9.7–10.5)	1,604	7.3 (7.0–7.7)	299	1.4 (1.2–1.5)
2011	10,159	738	7.3 (6.8–7.8)	375	3.7 (3.3–4.1)	162	1.6 (1.4–1.9)	224	2.2 (1.9–2.5)
2012	23,823	7,289	30.6 (30.0–31.2)	6,093	25.6 (25.0–26.1)	440	1.8 (1.7–2.0)	915	3.8 (3.6–4.1)
2013	20,192	3,998	19.8 (19.3–20.4)	1,840	9.1 (8.7–9.5)	1,813	9.0 (8.6–9.4)	519	2.6 (2.4–2.8)

* H1N1 + H3N2 + A/not subtyped.

N: Number of ILI+SARI records with samples tested for influenza (diagnosis might be + or -).

n: number of records within given category.

%: percentage within diagnosed cases = 100*n/N for each given category.

Acronyms: CI, confidence interval; ILI, influenza-like illness; SARI, severe acute respiratory infection.

The influenza season with the highest number of cases were April 2011–March 2012 and April 2012–March 2013 (Table 4, Figure 2) for influenza A and B respectively.

**Table 4.** Influenza-positive diagnoses, A, B or both, per ILI+SARI records, by influenza seasons.

	ILI+SARI records N	Influenza (A+B) diagnoses, n (%)	Influenza A diagnoses, n (%)	Influenza B diagnoses, n (%)
Jan 2010–Mar 2010	9,576	2,370 (24.7)	2,359 (24.6)	11 (0.1)
Apr 2010–Mar 2011	15,829	5,005 (31.6)	4,530 (28.6)	475 (3.0)
Apr 2011–Mar 2012	22,462	7,360 (32.8)	7,247 (32.3)	113 (0.5)
Apr 2012–Mar 2013	13,939	3,012 (21.6)	1,825 (13.1)	1,187 (8.5)
Apr 2013–Dec 2013	14,279	2,780 (19.5)	2,609 (18.3)	171 (1.2)

N: Number of ILI+SARI records with samples tested for influenza (diagnosis might be + or -).

n: number of records within given category.

%: percentage within diagnosed cases = 100*n/N for each given category.

Acronyms: ILI, influenza-like illness; SARI, severe acute respiratory infection.

### Regional distribution and clinical features

Influenza cases were distributed across all Mexican regions. Most influenza cases were reported in Mexico's Central region (Supplementary Table 2).

### Deaths

Amongst all 2010–2013 influenza cases, 944 deaths were recorded, almost all (98%, n = 926) had influenza A (Supplementary Table 5). Of the respective ILI+SARI patients, 230 patients died in 2010 of influenza A while only 57 died in 2011. A total of 18 (2%) patients died with influenza B, no patients died in 2010, whilst 10 died in 2013 (Supplementary Table 5).

## Discussion

This observational-retrospective database analysis used the data captured in the DGE database from 2010 to 2013, for investigating activity patterns and the demographic and clinical characteristics of influenza A and B occurrence. This is the first descriptive analysis to be completed on the influenza activity over 3 consecutive influenza seasons following the 2009 pandemic in Mexico. Since this pandemic, vaccination recommendations have been modified.

The current recommendation for vaccination is now that the annual vaccination should be administered to children ranging in age from 6 months to 4 years and adults of 60 years and older. As high-risk individuals now also include health workers, and individuals who are pregnant or morbidly obese. There is currently no data available on the effectiveness of the influenza vaccine in Mexico.^14^

However, studies have been completed on vaccine effectiveness in the US. In the 2017–2018 flu season, vaccine effectiveness against influenza A and influenza B was found overall to be about 36%, with most cases being from H3N2. Vaccine effectiveness against A(H1N1)pdm09 virus was 67%, and 42% against influenza B virus. The CDC continues to recommend vaccination since it can decrease hospitalization rates and prevent some illnesses.^15^

In 2010, the influenza season had an early start in August due to the epidemiological change that influenza pandemic posed in the epidemic profile of influenza. WHO GISRS data for Mexico, indicated that this early peak was mainly caused by A H3N2 virus activity.^13^ In the USA, the influenza 2010–2011 season started in November in from the southeastern states, and peaked nationally in February (2011).^16^^,^^17^ Similarly to Mexico, the predominant virus was the A H3N2 (62% of all influenza A cases), which co-circulated with the A H1N1 and influenza B viruses. In the USA, influenza B was the predominant virus in the southeastern states during November and December 2011 and the Victoria lineage was identified in 94% of all tested influenza B viruses (2011).^16^ In Canada, A H3N2 was the predominant virus in 2010–2011.^18^

According to the GISRS data, the influenza peak recorded in 2012, consisted mainly of activity of the recrudescent pandemic A (H1N1)pdm09 virus (2012).^16^ Chowell and colleagues (2012) analyzed data from 760 A H1N1 positive respiratory-infection patients that were hospitalized in Mexico between December 2011 and February 2012^8^. They found that this fourth wave of the pandemic virus caused more hospitalizations than the previous three waves in 2009. They also reported a shift towards older age distribution of hospitalizations and deaths.^6^ In our analysis, one third of all influenza cases were individuals hospitalized because of their illness. Given the reported peak of influenza activity in January-February 2012, we hypothesize that most of these hospitalizations were caused by this recrudescent pandemic wave. There was no such pandemic wave in the USA, in which the A H3N2 type was predominant (2010-2013).^16^^,^^19^

The highest activity for influenza B virus was recorded in the influenza season that followed the peak of influenza A activity. We know, from the analysis of influenza prevalence in the USA over a period of 30 years, that influenza B prevalence is lower at periods with higher levels of influenza A prevalence.^20^ During or shortly following these periods, a shift in the dominant B lineage occurs.^20^ Immune selection may determine the evolutionary dynamics of influenza B virus and alter the lineage dominance.^20^ Increasing immunity to the dominant lineage reduces its frequency but also leaves room for other lineages to become dominant.^20^ Which lineage will predominantly circulate each season cannot be predicted with certainty, which makes developing an influenza vaccine challenging. For example, in half of the influenza seasons in the USA since 2000, and in one quarter of influenza seasons examined in 26 countries between 2000 and 2013, the dominant lineage did not match with the one included in the seasonal vaccine.^21^^,^^22^ However the vaccine can still provide protection against some of the co-circulating viruses present during a particular influenza season.^15^ The latest available GISRS data (season 2016–2017) for Mexico showed that B/Yamagata lineage was more common than B/Victoria lineage. The recently isolated B/Yamagata/16/88 lineage viruses are antigenically and genetically related to B/Phuket/3073/2013.^23^ Therefore, a B/Phuket/3073/2013-like virus not included in the commonly used trivalent vaccines is one of the components of the quadrivalent vaccine recommended for use in the 2016–2017 Northern hemisphere influenza season.^23^

In agreement with previously published findings, from across the globe, school-age children and adolescents have been found to have a higher susceptibility to influenza B virus.^8^^,^^22^^,^^24-27^ School-age children and adolescents are the first populations to be affected by influenza infection.^28^ Children need more time to acquire natural immunity to influenza B compared to influenza A. In a population-based study in the Netherlands 72% of children aged seven years had developed antibodies to influenza B and 100% to influenza A.^29^ Interestingly, several studies have shown that the Victoria lineage affects younger people, while Yamagata is more evenly distributed across age groups.^27^^,^^30-33^ In addition, the antibodies against both B lineages did not cross-react.^30^

Some previously published studies on the epidemiology of influenza in Mexico, used data from hospitalized influenza cases^1^^,^^5^^,^^8^^,^^9^ or more frequently studies used data from the prospective surveillance system implemented in 2009 by the Mexican Institute for Social Security (IMSS for Spanish acronym).^1^^,^^3^^,^^7-9^ IMSS collects data from 1,099 primary clinics and 259 hospitals across Mexico, covering 40% of the Mexican population,^3^^,^^8^ while excluding the lower socio-economic population.^9^ Hence, studies using the IMSS database have an inherent selection bias towards hospitalized patients and private sector employees with middle to upper socio-economic background. To our knowledge, the present study is the first to use DGE data involving all Mexican citizens, containing information on medically-attended visits to general practitioner offices as well as hospitalizations or not, irrespective of medical insurance type or socio-economic status.

A key factor for successful influenza control and effective vaccination strategies is an effective and reliable national surveillance system.^34^ The documentation of virus activity that is performed via such systems offers a unique opportunity to characterize seasonal patterns, develop reliable estimates, and identify parameters affecting preparedness for future epidemics.^35^ An analysis on USA Medicare and Medicaid records, showed firstly that seasons with early influenza activity tend to produce more influenza cases, and secondly that the timing and absolute intensity of influenza activity may serve as a proxy for estimating the time of year with the highest influenza incidence. Such information can improve preparedness for management of hospitalizations and comorbidities.^35^ Since 2004, Canada sentinel surveillance system has incorporated vaccine effectiveness data.^18^ Based on this dataset, vaccine effectiveness studies have been published providing highly informative results that may guide decisions in reformulating vaccines in terms of composition and in improving disease protection.^18^^,^^36-38^ Current recommendations for influenza virus vaccinations in Mexico include the administration of a trivalent vaccine to children and high-risk patients. However, our analysis indicates that younger adults are also vulnerable to both influenza A and B viruses and that only one out of five patients with ILI had received a vaccination. In addition, for an effective vaccination strategy, we need to reduce the probability of having a mismatch between the seasonal vaccine and the dominant influenza B lineage. Although our dataset did not contain lineage information, DGE has now started to characterize B strains, which will provide valuable information for a successful implementation of the vaccination strategy. A limitation that we had was that there is no currently available data on the effectiveness of the vaccination program in Mexico.

Influenza surveillance in Mexico is based on sentinel sites and as DGE is a secondary database, underreporting is expected, as DGE is only a sample of all ILI/SARI cases over the National Healthcare System, hence the reason ILI+SARI were reported together. We state that this is a limiting factor of the analysis as patients may be recorded twice. However, this limitation is expected to be counterbalanced by the large sample size, representative of the Mexican population. In addition, a large proportion of influenza A cases were not subtyped, and detail on B lineages were not available within the data provided for this analysis. A more sensitive description of the within seasonal variation would have allowed us to identify times of predominance of one virus over the other for example. However, this data was not available and should be identified as an area to consider for future work. Lastly, our analysis should have ideally covered a five season period from April 2009 to March 2014 (based on the WHO defined influenza seasons^23^) to help characterize potential seasonal patterns, however this data was not available, therefore we characterized the data based on observed seasons within the data we had available. Whilst this is not optimal it is unlucky to affect our findings due to seasonal distribution peaks throughout each period.

## Conclusions

Using a validated epidemiological surveillance system, representative of the Mexican population, this study provides unique meaningful information on the influenza activity in Mexico over three consecutive influenza seasons. This analysis confirms that influenza is an important respiratory pathogen for children and adults in Mexico despite the fact that an influenza vaccination program has been implemented since 2004. School-age children and adolescents were more prone to influenza B infection while younger adults were susceptible to both influenza A and B viruses. Over the three analyzed seasons, influenza B virus continuously co-circulated with influenza A virus. However, lineage characterization for influenza B viruses was not available, such description is needed to strengthen the knowledge of influenza B strains characteristics for immunization strategies in the country. Increased knowledge of lineage characteristics would lead to improved vaccines.

## Methods

### Study design and setting

The study was conducted in accordance with all applicable ethics requirements, including patient privacy and the guiding principles of the International Ethical Guidelines for Epidemiological Studies. Due to the nature of the study (i.e., retrospective by nature) and the anonymisation process prior to data extraction, as per research standards and local regulations, no ethics committee approval was required to conduct the analysis. The dataset was anonymised before data extraction, so it was not possible to make a link between the extracted data and specific individuals. Therefore, no informed consent was required from patients. Regarding the surveillance activities in the country, participants agreed with the terms of the standardised healthcare and the subsequent sample analysis and report. The data were analysed between December 2014 and November 2015.

### Population data

All January 2010–December 2013 DGE´s ILI and SARI records were eligible for inclusion in the analysis dataset.

### Description of the DGE database

Since 2006, a sentinel surveillance program has continued to be conducted in Mexico in order to collect data on influenza. The Weekly Report System for New Cases (SUAVE for Spanish acronym) and the Influenza Monitoring Healthcare Units (USMI for Spanish acronym), were developed to support this program. SUAVE is a weekly, routine, passive, population-based surveillance system, based on the International Classification of Diseases (ICD) coding system, where 50 diagnoses are broken down into 13 disease groups.^39^ The USMIs consist of 583 designated primary healthcare facilities and general hospitals (including second and third-level hospitals) found throughout Mexico. Their role is to use systematic and standardized procedures to collect samples of persons with ILIs.^40^ The USMI conducts sentinel surveillance according to the World Health Organisation (WHO) recommendations. The USMI surveillance system is synonymous with the US and Canadian systems.^39^ Since October 2009, all confirmed influenza cases: ILI, and SARI are registered via SUAVE and USMI in a unique informative platform. From this platform, the information is centralised at the DGE national surveillance program database.^12^ Before being entered into SUAVE and UNMIs information is checked locally for consistency where the number of cases must be equal or higher in the SUAVE due to the more general symptom recording procedure of SUAVE.

Throat and nasopharyngeal swab samples were collected and tested for the presence of influenza A and B viruses in adults and infants (including very young children), respectively. Reverse transcriptase-polymerase chain reaction (rt-PCR) analysis was used to confirm cases,^39^ and results were recorded in the database.

### Case definitions

ILI: Fever (≥38°C), cough and headache accompanied by one or more of the following symptoms: rhinorrhea, coryza, arthralgia, myalgia, fatigue, sore throat, chest pain, abdominal pain, nasal congestion and diarrhea. For patients under five years of age, signs of irritability were recorded instead of headache.

SARI: Shortness of breath, history of fever (≥38°C) and cough, and any of the following symptoms: malaise, chest pain, tachypnea or acute respiratory distress syndrome. Influenza-related pneumonia and chronic disease exacerbations were included.

Influenza confirmed case: Patients who tested positive (in rt-PCR) for influenza.

### Objectives

This analysis aimed to primarily describe influenza A and B cases, by age group and A-subtype/strain B-lineage during the period analysed.

Additionally, we aimed to describe the number of ILI cases attributable to influenza A and B, the regional distribution of influenza A and B cases, the frequency of clinical features and outcomes (clinical symptoms, duration of illness, complications) experienced by patients with ILI/SARI who were positive for influenza A or B, and finally the proportion of influenza cases among ILI and SARI cases.

Age at date of symptom onset was computed as the difference between the date of onset of the first symptom and the date of birth, and was measured in years for patients older than one year, and in months for patients younger than one year.

The age groups analysed were: 0–4, 5–17, 18–49, 50–64, and ≥65 years.

### Data collection and procedures

All influenza A and B, ILI and SARI cases in the DGE database were extracted to a Microsoft Excel file and converted into Statistical Analysis System datasets for analysis. Demographic data, date of symptom onset, geographic region (Center, Center-West, North, and South-Southeast), clinical features and outcomes (clinical symptoms, duration of illness, complications) for all patients with ILI caused by influenza A and B were extracted and anonymised by DGE personnel and then transferred to the sponsor. The data were then analysed.

### Statistical analyses

Descriptive statistics were used including frequency tables generated for categorical variables. The mean, median, and standard deviation were calculated for continuous data. The distribution of influenza cases by month and year was tabulated by their respective frequencies and percentages, overall and by age group and strain subtype/lineage. The proportion of cases attributable to influenza A and B was calculated with exact 95% confidence intervals (CI) for the overall sample and by age and subtype/lineage.

The statistical analyses were performed using the Statistical Analysis Systems version 9.2, the Drug and Development web portal version 3.5, and Microsoft Excel 2007.

## Supplementary Material

KHVI_A_1456281_Supplemental.docx

## Abbreviations

CI: Confidence Interval
DGE: Directorate General of Epidemiology
GISRS: Global Influenza Surveillance and Response System
ILI: Influenza-Like Illness
IMSS: Mexican Institute of Social Security
rt-PCR: reverse transcriptase Polymerase Chain Reaction
SARI: Severe Acute Respiratory Infection
SUAVE: Weekly Report System for New Cases
USMI: Influenza Monitoring Healthcare Units
WHO: World Health Organisation
