# Supplementary material for: The burden of influenza A and B in Mexico from the year 2010 to 2013: An observational, retrospective, database study, on records from the Directorate General of Epidemiology database
Source: Hum Vaccin Immunother. 2018 May 10;14(8):1890–8. doi: 10.1080/21645515.2018.1456281 (PMC6149840; doi:10.1080/21645515.2018.1456281)
Supplement: KHVI_A_1456281_Supplemental.docx [file khvi-14-08-1456281-s001.docx]

SUPPLEMENTARY MATERIAL

**Supplementary Table 1.** Occurrence of influenza cases by year, within the ILI+SARI, ILI, and SARI records with influenza diagnosis, 2010–2013

|  | ILI+SARI records | | | ILI records | | | SARI records | | |
| --- | --- | --- | --- | --- | --- | --- | --- | --- | --- |
|  | N | Influenza cases | | N | Influenza cases | | N | Influenza cases | |
|  |  | (n) | % (95%CI) |  | (n) | % (95%CI) |  | (n) | % (95%CI) |
| 2010-2013 | 76,085 | 20,527 | 27.0 (26.7–27.3) | 38,876 | 12,254 | 31.5 (31.1–32.0) | 37,209 | 8,273 | 22.2 (21.8–22.7) |
| 2010 | 21,911 | 6,844 | 31.2 (30.6–31.9) | 12,012 | 3,886 | 32.4 (31.5–33.2) | 9,899 | 2,958 | 29.9 (29.0–30.8) |
| 2011 | 10,159 | 962 | 9.5 (8.9–10.1) | 5,505 | 608 | 11.0 (10.2–11.9) | 4,654 | 354 | 7.6 (6.9–8.4) |
| 2012 | 23,823 | 8,204 | 34.4 (33.8–35.0) | 12,738 | 5,244 | 41.2 (40.3–42.0) | 11,085 | 2,960 | 26.7 (25.9–27.5) |
| 2013 | 20,192 | 4,517 | 22.4 (21.8–23.0) | 8,621 | 2,516 | 29.2 (28.2–30.2) | 11,571 | 2,001 | 17.3 (16.6–18.0) |

N: Number of records with samples tested for influenza (diagnosis might be + or –)
n: number influenza- positive samples within given category
%: percentage within diagnosed cases = 100*n/N for each given category
Acronyms: CI, confidence interval; ILI, influenza-like illness; SARI, severe acute respiratory infection;

**Supplementary Table 2.** Geographical distribution of influenza A and B, 2010–2013.

|  |  | Influenza-positive | | Influenza A | | Influenza B | |
| --- | --- | --- | --- | --- | --- | --- | --- |
|  | N | n | % (95%CI) | n | % (95%CI) | n | % (95%CI) |
| All Regions | 76,085 | 20,527 | 27.0 (26.7–27.3) | 18,570 | 24.4 (24.1–24.7) | 1,957 | 2.6 (2.5–2.7) |
| Center | 23,308 | 6,377 | 27.4 (26.8–27.9) | 5,786 | 24.8 (24.3–25.4) | 591 | 2.5 (2.3–2.7) |
| Center–West | 17,355 | 4,442 | 25.6 (24.9–26.3) | 4,041 | 23.3 (22.7–23.9) | 401 | 2.3 (2.1–2.5) |
| North | 15,073 | 4,963 | 32.9 (32.2–33.7) | 4,416 | 29.3 (28.6–30.0) | 547 | 3.6 (3.3–3.9) |
| South–Southeast | 20,349 | 4,745 | 23.3 (22.7–23.9) | 4,327 | 21.3 (20.7–21.8) | 418 | 2.1 (1.9–2.3) |

N: Number of ILI+SARI records with samples tested for influenza (diagnosis might be + or -)
n: number of records within given category
%: percentage within diagnosed cases 100*n/N for each given category
Acronyms: CI, confidence interval; ILI, influenza-like illness; SARI, severe acute respiratory infection

**Supplementary Table 3.** Occurrence of influenza A and B by year, within the ILI records with influenza diagnosis, 2010–2013

|  | ILI records | ILI Influenza A samples | | ILI Influenza A H1N1 | | ILI Influenza A H3N2 | | ILI Influenza B | |
| --- | --- | --- | --- | --- | --- | --- | --- | --- | --- |
|  | N | n | % (95%CI) | n | % (95%CI) | n | % (95%CI) | n | % (95%CI) |
| 2010–2013 | 38,876 | 10,764 | 27.7 (27.2–28.1) | 6,009 | 15.5 (15.1–15.8) | 2,742 | 7.1 (6.8–7.3) | 1,490 | 3.8 (3.6–4.0) |
| 2010 | 12,012 | 3,625 | 30.2 (29.4–31.0) | 1,207 | 10.0 (9.52–10.6) | 1,086 | 9.0 (8.5–9.6) | 261 | 2.2 (1.9–2.4) |
| 2011 | 5,505 | 416 | 7.6 (6.9–8.3) | 202 | 3.7 (3.2–4.2) | 138 | 2.5 (2.1–3.0) | 192 | 3.5 (3.0–4.0) |
| 2012 | 12,738 | 4,553 | 35.7 (34.9–36.6) | 3,839 | 30.1 (29.3–30.9) | 299 | 2.3 (2.1–2.6) | 691 | 5.4 (5.0–5.8) |
| 2013 | 8,621 | 2,170 | 25.2 (24.3–26.1) | 761 | 8.8 (8.2–9.4) | 1,219 | 14.1 (13.4–14.9) | 346 | 4.0 (3.6–4.4) |

N: Number of ILI records with samples tested for influenza (diagnosis might be + or –)
n: number of records within given category
%: percentage within diagnosed cases 100*n/N for each given category
Acronyms: CI, confidence interval; ILI, influenza-like illness

**Supplementary Table 4.** Clinical manifestations, outcomes, and comorbidities of the ILI influenza A and B cases, by year 2010, 2011, 2012, and 2013

|  | 2010 | | 2011 | | 2012 | | 2013 | |
| --- | --- | --- | --- | --- | --- | --- | --- | --- |
|  | Influenza A | Influenza B | Influenza A | Influenza B | Influenza A | Influenza B | Influenza A | Influenza B |
|  | n (%) | n (%) | n (%) | n (%) | n (%) | n (%) | n (%) | n (%) |
| **Clinical manifestations** |  |  |  |  |  |  |  |  |
| Sudden onset of symptoms | 2,786 (76.9) | 230 (88.1) | 318 (76.4) | 152 (79.2) | 3,643 (80.0) | 538 (77.9) | 1,640 (75.6) | 282 (81.5) |
| Fever | 3,443 (95.0) | 251 (96.2) | 396 (95.2) | 186 (96.9) | 4,356 (95.7) | 675 (97.7) | 2,043 (94.1) | 321 (92.8) |
| Malaise | 2,907 (80.2) | 207 (79.3) | 337 (81.0) | 144 (75.0) | 3,740 (82.1) | 518 (75.0) | 1,686 (77.7) | 273 (78.9) |
| Cough | 3,462 (95.5) | 251 (96.2) | 395 (95.0) | 179 (93.2) | 4,363 (95.8) | 663 (95.9) | 2,005 (92.4) | 315 (91.0) |
| Rhinorrhea | 2,816 (77.7) | 206 (78.9) | 314 (75.5) | 153 (79.7) | 3,579 (78.6) | 550 (79.6) | 1,617 (74.5) | 264 (76.3) |
| Headache | 3,041 (83.9) | 211 (80.8) | 352 (84.6) | 162 (84.4) | 3,948 (86.7) | 604 (87.4) | 1,748 (80.6) | 280 (80.9) |
| Polypnea | 694 (19.1) | 31 (11.9) | 98 (23.6) | 30 (15.6) | 888 (19.5) | 73 (10.6) | 458 (21.1) | 65 (18.8) |
| Dyspnea | 1,459 (40.2) | 85 (32.6) | 179 (43.0) | 65 (33.9) | 1,973 (43.3) | 193 (27.9) | 988 (45.5) | 122 (35.3) |
| Vomiting | 481 (13.3) | 32 (12.3) | 59 (14.2) | 24 (12.5) | 687 (15.1) | 115 (16.6) | 313 (14.4) | 55 (15.9) |
| Myalgia | 2,593 (71.5) | 179 (68.6) | 292 (70.2) | 134 (69.8) | 3,484 (76.5) | 484 (70.0) | 1,550 (71.4) | 253 (73.1) |
| Arthalgia | 2,392 (66.0) | 163 (62.5) | 275 (66.1) | 123 (64.1) | 3,256 (71.5) | 421 (60.9) | 1,420 (65.4) | 217 (62.7) |
| Irritability | 1,167 (32.2) | 91 (34.9) | 136 (32.7) | 64 (33.3) | 1,805 (39.6) | 231 (33.4) | 772 (35.6) | 143 (41.3) |
| Odynophagia | 2,114 (58.3) | 152 (58.2) | 221 (53.1) | 133 (69.3) | 2,642 (58.0) | 410 (59.3) | 1,176 (54.2) | 217 (62.7) |
| Abdominal pain | 802 (22.1) | 62 (23.8) | 89 (21.4) | 37 (19.3) | 1,050 (23.1) | 130 (18.8) | 451 (20.8) | 82 (23.7) |
| Diarrhea | 545 (15.0) | 46 (17.6) | 59 (14.2) | 23 (12.0) | 591 (13.0) | 78 (11.3) | 244 (11.2) | 50 (14.5) |
| Conjunctivitis | 1,032 (28.5) | 84 (32.2) | 95 (22.8) | 56 (29.2) | 1,344 (29.5) | 173 (25.0) | 526 (24.2) | 111 (32.1) |
| Chest pain | 1,474 (40.7) | 105 (40.2) | 185 (44.5) | 72 (37.5) | 2,189 (48.1) | 241 (34.9) | 914 (42.1) | 128 (37.0) |
| Cyanosis | 246 (6.8) | 10 (3.8) | 30 (7.2) | 6 (3.1) | 229 (5.0) | 19 (2.7) | 124 (5.7) | 13 (3.8) |
| Chills | 2,470 (68.1) | 169 (64.8) | 294 (70.7) | 129 (67.2) | 3,266 (71.7) | 449 (65.0) | 1,396(64.3) | 220 (63.6) |
| **Co-morbitities** |  |  |  |  |  |  |  |  |
| Diabetes | 210 (5.8) | 6 (2.3) | 33 (7.9) | 6 (3.1) | 213 (4.7) | 28 (4.1) | 191 (8.8) | 14 (4.0) |
| COPD | 63 (1.7) | 2 (0.8) | 10 (2.4) | 1 (0.5) | 63 (1.4) | 12 (1.7) | 52 (2.4) | 6 (1.7) |
| Asthma | 186 (5.1) | 18 (6.9) | 14 (3.4) | 7 (3.6) | 236 (5.2) | 27 (3.9) | 119 (5.5) | 20 (5.8) |
| Immunosupression | 73 (2.0) | 5 (1.9) | 8 (1.9) | 0 (0.0) | 81 (1.8) | 6 (0.9) | 46 (2.1) | 11 (3.2) |
| Hypertension | 239 (6.6) | 5 (1.9) | 43 (10.3) | 6 (3.1) | 286 (6.3) | 29 (4.2) | 186 (8.6) | 18 (5.2) |
| HIV / AIDS | 35 (1.0) | 6 (2.3) | 7 (1.7) | 1 (0.5) | 36 (0.8) | 8 (1.2) | 14 (0.6) | 3 (0.9) |
| Cardiovascular disease | 64 (1.8) | 1 (0.4) | 9 (2.2) | 3 (1.6) | 74 (1.6) | 7 (1.0) | 47 (2.2) | 11 (3.2) |
| Obesity | 272 (7.5) | 12 (4.6) | 44 (10.6) | 10 (5.2) | 447 (9.8) | 33 (4.8) | 238 (11.0) | 25 (7.2) |
| Smoking | 283 (7.8) | 11 (4.2) | 25 (6.0) | 8 (4.2) | 389 (8.5) | 29 (4.2) | 169 (7.8) | 14 (4.0) |
| Chronic renal failure | 46 (1.3) | 2 (0.8) | 8 (1.9) | 1 (0.5) | 64 (1.4) | 6 (0.9) | 42 (1.9) | 4 (1.2) |
| Pregmancy | 124 (6.0) | 5 (3.3) | 12 (5.2) | 7 (7.1) | 141 (5.5) | 10 (2.7) | 76 (5.9) | 12 (5.9) |
| **Outcome** |  |  |  |  |  |  |  |  |
| Death | 60 (1.7) | 0 (0.0) | 10 (2.4) | 0 (0.0) | 59 (1.4) | 1 (0.1) | 79 (3.6) | 4 (1.2) |
| Discharged | 204 (5.8) | 5 (2.0) | 37 (9.0) | 4 (2.2) | 262 (6.0) | 20 (2.9) | 249 (11.5) | 33 (9.5) |
| Follow-up | 773 (22.1) | 43 (16.9) | 79 (19.3) | 49 (26.6) | 1,445 (33.2) | 138 (20.1) | 498 (22.9) | 96 (27.7) |
| In treatment | 1,595 (45.5) | 156 (61.4) | 158 (38.6) | 97 (52.7) | 1,926 (44.3) | 436 (63.4) | 1,000 (46.1) | 173 (50.0) |
| Non severe cases | 622 (17.7) | 45 (17.7) | 90 (22.0) | 20 (10.9) | 490 (11.3) | 76 (11.0) | 238 (11.0) | 25 (7.2) |
| Severe cases | 249 (7.1) | 4 (1.6) | 35 (8.6) | 14 (7.6) | 155 (3.6) | 17 (2.5) | 104 (4.8) | 14 (4.0) |
| Transferred | 2 (0.1) | 1 (0.4) | 0 (0.0) | 0 (0.0) | 10 (0.2) | 0 (0.0) | 2 (0.1) | 1 (0.3) |
| Missing | 120 | 7 | 7 | 8 | 206 | 3 | - | - |

n: number of episode in a given category
%: n/ number of episodes in a given category*100
Acronyms: AIDS, acquired immune deficiency syndrome; COPD, chronic obstructive pulmonary disease; HIV, human immunodeficiency virus; ILI, influenza-like illness;

**Supplementary Table 5.** Number of deaths recorded every year between 2010–2013, within the ILI and SARI populations by influenza virus A and B diagnosis.

|  | ILI | | | SARI | | | | ILI + SARI | | | | |  |
| --- | --- | --- | --- | --- | --- | --- | --- | --- | --- | --- | --- | --- | --- |
|  | Influenza A | Influenza B | Influenza (A + B) | Influenza A | Influenza B | Influenza (A + B) | Influenza A | | Influenza B | | Influenza (A + B) | | |
| 2010–2013 | 208 | 5 | 213 | 718 | 13 | 731 | | 926 | | 18 | | 944 |  |
| 2010 | 60 | 0 | 60 | 170 | 0 | 170 | | 230 | | 0 | | 230 |  |
| 2011 | 10 | 0 | 10 | 47 | 1 | 48 | | 57 | | 1 | | 58 |  |
| 2012 | 59 | 1 | 60 | 256 | 6 | 262 | | 315 | | 7 | | 322 |  |
| 2013 | 79 | 4 | 83 | 245 | 6 | 251 | | 324 | | 10 | | 334 |  |

Acronyms: ILI, influenza-like illness; SARI, severe acute respiratory infection
